# Bis(μ-2-hydroxy­benozato)-κ^3^ *O*,*O*′:*O*′;κ^3^ *O*:*O*,*O*′-bis­[(2-hydroxy­benozato-κ^2^ *O*,*O*′)(1,10-phenanthroline-κ^2^ *N*,*N*′)cadmium(II)]

**DOI:** 10.1107/S1600536808033886

**Published:** 2008-10-25

**Authors:** Qing-Yuan Shi, Zhi-Cheng Li, Zong-Sheng Cheng, Jing-Bo Tan, Jia-Lun Liu

**Affiliations:** a School of Chemistry and Environment, South China Normal University, Guangzhou 510006, People’s Republic of China

## Abstract

The dinuclear title compound, [Cd_2_(C_7_H_5_O_3_)_4_(C_12_H_8_N_2_)_2_], is located on a crystallographic rotation twofold axis. The two Cd^II^ ions are connected by two tridentate bridging 2-hydroxy­benzoate anions. Each Cd^II^ ion is seven-coordinated by five O atoms from three 2-hydroxy­benzoate ligands and two N atoms from 1,10-phenanthroline. The 2-hydroxy­benzoate mol­ecules adopt two kinds of coordination mode, bidentate chelating and tridentate bridging–chelating. Intra­molecular hydrogen bonds between hydr­oxy and carboxyl­ate groups from 2-hydroxy­benzoate groups and π–π stacking interactions between parallel 1,10-phenanthroline ligands [centroid–centroid distances = 3.707 (3) and 3.842 (3) Å] are observed. Furthermore, adjacent benzene rings from 2-hydroxy­benzoate ligands are involved in π–π inter­actions with inter­planar distances of 3.642 (3) Å, thereby forming a chain along the *a* axis direction.

## Related literature

For general background, see: Horike *et al.* (2007 ▶); Humphrey *et al.* (2007 ▶); Sudik *et al.* (2005 ▶); Zhang *et al.* (2008 ▶). For related structures, see: Du *et al.* (2007 ▶); Pan *et al.* (2006 ▶); Tomas *et al.* (2006 ▶). For related literature, see: Tong *et al.* (1999 ▶)
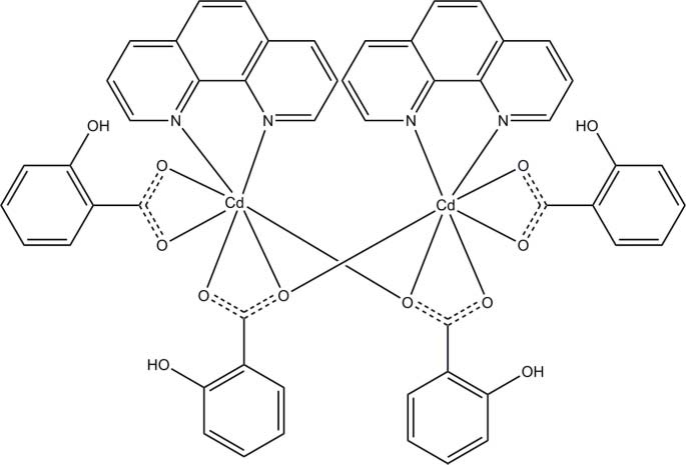


## Experimental

### 

#### Crystal data

[Cd_2_(C_7_H_5_O_3_)_4_(C_12_H_8_N_2_)_2_]
*M* *_r_* = 1133.65
Monoclinic, 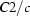

*a* = 27.9391 (19) Å
*b* = 10.3078 (7) Å
*c* = 20.468 (2) Å
β = 130.770 (1)°
*V* = 4464.2 (6) Å^3^
*Z* = 4
Mo *K*α radiation
μ = 1.03 mm^−1^
*T* = 298 (2) K
0.30 × 0.25 × 0.18 mm

#### Data collection

Bruker APEXII CCD area-detector diffractometer
Absorption correction: multi-scan (*SADABS*; Sheldrick, 1996 ▶) *T*_min_ = 0.748, *T*_max_ = 0.837
11963 measured reflections
4390 independent reflections
3671 reflections with *I* > 2σ(*I*)
*R*_int_ = 0.027

#### Refinement

*R*[*F* ^2^ > 2σ(*F* ^2^)] = 0.026
*wR*(*F* ^2^) = 0.067
*S* = 1.06
4390 reflections
316 parameters
H-atom parameters constrained
Δρ_max_ = 0.25 e Å^−3^
Δρ_min_ = −0.36 e Å^−3^

### 

Data collection: *APEX2* (Bruker, 2004 ▶); cell refinement: *SAINT* (Bruker, 1999 ▶); data reduction: *SAINT*; program(s) used to solve structure: *SHELXS97* (Sheldrick, 2008 ▶); program(s) used to refine structure: *SHELXL97* (Sheldrick, 2008 ▶); molecular graphics: *SHELXTL* (Sheldrick, 2008 ▶) and *ORTEP-3* (Farrugia, 1997 ▶); software used to prepare material for publication: *SHELXTL*.

## Supplementary Material

Crystal structure: contains datablocks I, global. DOI: 10.1107/S1600536808033886/zl2145sup1.cif

Structure factors: contains datablocks I. DOI: 10.1107/S1600536808033886/zl2145Isup2.hkl

Additional supplementary materials:  crystallographic information; 3D view; checkCIF report

## Figures and Tables

**Table 1:** Hydrogen-bond geometry (Å, °)

*D*—H⋯*A*	*D*—H	H⋯*A*	*D*⋯*A*	*D*—H⋯*A*
O6—H6*A*⋯O4	0.82	1.86	2.579 (3)	146
O3—H3*A*⋯O2	0.82	1.87	2.576 (2)	143

**Fig. 1.**
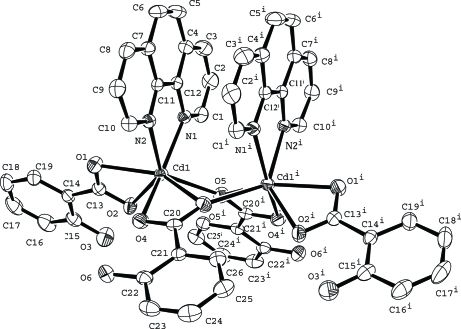
An ORTEP-3 (Farrugia, 1997) plot of the title compound with displacement ellipsoids at the 30% probability level. All H atoms are omitted for clarity.

**Fig. 2.**
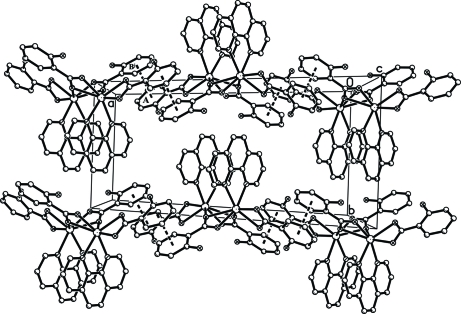
A packing diagram of the title compound, showing a one-dimensional chain-like structure generated by the intermolecular π-π interactions. All H atoms are omitted for clarity. [Symmetry codes: (j): 1/2 + x, 1/2 - y, 1/2 + z].

## supplementary crystallographic information

### Comment

Transition metal complexes with substituted benzoate ligands have attracted wide attention in past decades, owing to their variable high-dimensional architectures and potential applications for gas absorption and separation, and catalysis, *etc* [Du *et al.*, 2007; Horike *et al.*, 2007; Humphrey *et al.*, 2007; Pan *et al.*, 2006; Sudik *et al.*, 2005; Tomas *et al.*, 2006; Zhang *et al.*, 2008]. Herein, we report the synthesis and crystal structure of the title compound, obtained by the reaction of Sm(NO_3_)_3_, Cd(CH_3_COO)_2_, 1,10-phenanthroline and 2-hydroxybenzoic acid. No samarium was however incorporated in the crystals isolated and the title compound is a dinuclear Cd^II^ complex of 2-hydroxybenzoic acid and 1,10-phenanthroline. A perspective view of the complex, showing the atomic numbering scheme, is depicted in Fig. 1. Each Cd^II^ is seven-coordinated by five oxygen atoms from three 2-hydroxybenzoate ligands, and two nitrogen atoms from 1,10-phenanthroline, and the coordination geometry around the Cd^II^ ion may be described as a distorted mono-capped trigonal prism. Two adjacent Cd^II^ units are connected by two bridging 2-hydroxybenzoate anions to generate a dinuclear complex. The 2-hydroxybenzoate molecules adopt two kinds of coordination modes, bidentate chelating and tridentate bridging-chelating. The inequivalence between the mono and bidentate bridging oxygen atoms is evident from the Cd—O bond distances: the Cd—O distances of the bridging oxygen atoms are longer than those of the monodentate oxygen atoms: Cd1—O5 is 2.421 (2) Å and Cd1—O5^i^ is 2.491 (2) Å, while Cd1—O1, Cd—O2 and Cd—O4 are 2.399 (2), 2.327 (2) and 2.363 (2) Å, respectively (symmetry code: (i): -*x*, *y*, 1/2 - *z*).

Intramolecular hydrogen bonds between hydroxyl and carboxylate groups from the 2-hydroxybenzoates [O6···O4 = 2.579 (3) Å and O3···O2 = 2.576 (2) Å, Table 1] and π-π stacking attractions between parallel 1,10-phenanthroline ligands [centroid to centroid distances: 3.707 (3) and 3.842 (3) Å] are clearly observed in this complex, which may contribute to its stability.

Furthermore, adjacent phenyl rings from 2-hydroxybenzoate ligands are also involved in π-π stacking interactions by partial overlap of π-electron densities (Tong *et al.*, 1999). The centroid-centroid separation between rings A (atoms C14—C19) and B*^j^* [atoms C21—C26; symmetry code: (*j*): 1/2 + *x*, 1/2 - *y*, 1/2 + *z*] is 3.642 (3) Å. Considering these π-π intermolecular attractions, they imply the formation of a one-dimensional chain along the direction of the a-axis. (Fig. 2).

### Experimental

A sample of Sm(NO_3_)_3_.6H_2_O (0.090 g, 0.20 mmol), Cd(CH_3_COO)_2_.2H_2_O (0.052 g, 0.20 mmol), 2-hydroxybenzoic acid (0.070 g, 0.50 mmol), 1,10-phenanthroline (0.036 g, 0.20 mmol) and distilled water (8 ml) were mixed in a 15 ml Teflon-lined stainless steel vessel and the pH value was adjusted to about 5 with NaOH. Then, the mixture was heated to 393 K under autogenous pressure for 48 h, and cooled slowly to room temperature. Colorless block-like crystals suitable for X-ray single-crystal diffraction analysis were obtained by filtration and washed with distilled water and ethanol.

### Refinement

All H atoms were placed in calculated positions and were allowed to ride on their parent atoms; C—H = 0.93 (aromatic C—H) and O—H = 0.82 (hydroxyl) Å; *U*_iso_(H) = 1.2 *U*_eq_ (C) and *U*_iso_ (H) = 1.5 *U*_eq_ (O).

### Crystal data

**Table:**

[Cd_2_(C_7_H_5_O_3_)_4_(C_12_H_8_N_2_)_2_]	*F*(000) = 2272
*M**_r_* = 1133.65	*D*_x_ = 1.687 Mg m^−^^3^
Monoclinic, *C*2/*c*	Mo *K*α radiation, λ = 0.71073 Å
Hall symbol: -C 2yc	Cell parameters from 4082 reflections
*a* = 27.9391 (19) Å	θ = 1.9–27.4°
*b* = 10.3078 (7) Å	µ = 1.03 mm^−^^1^
*c* = 20.468 (2) Å	*T* = 298 K
β = 130.770 (1)°	Block, colorless
*V* = 4464.2 (6) Å^3^	0.30 × 0.25 × 0.18 mm
*Z* = 4	

### Data collection

**Table:**

Bruker SMART APEXII CCD area-detector diffractometer	4390 independent reflections
Radiation source: fine-focus sealed tube	3671 reflections with *I* > 2σ(*I*)
graphite	*R*_int_ = 0.027
φ and ω scans	θ_max_ = 26.0°, θ_min_ = 1.9°
Absorption correction: multi-scan (SADABS; Sheldrick, 1996)	*h* = −34→23
*T*_min_ = 0.748, *T*_max_ = 0.837	*k* = −12→12
11963 measured reflections	*l* = −18→25

### Refinement

**Table:**

Refinement on *F*^2^	Primary atom site location: structure-invariant direct methods
Least-squares matrix: full	Secondary atom site location: difference Fourier map
*R*[*F*^2^ > 2σ(*F*^2^)] = 0.026	Hydrogen site location: inferred from neighbouring sites
*wR*(*F*^2^) = 0.067	H-atom parameters constrained
*S* = 1.06	*w* = 1/[σ^2^(*F*_o_^2^) + (0.0314*P*)^2^ + 1.2193*P*] where *P* = (*F*_o_^2^ + 2*F*_c_^2^)/3
4390 reflections	(Δ/σ)_max_ = 0.001
316 parameters	Δρ_max_ = 0.25 e Å^−^^3^
0 restraints	Δρ_min_ = −0.36 e Å^−^^3^

### Special details

**Table:**

Geometry. All e.s.d.'s (except the e.s.d. in the dihedral angle between two l.s. planes) are estimated using the full covariance matrix. The cell e.s.d.'s are taken into account individually in the estimation of e.s.d.'s in distances, angles and torsion angles; correlations between e.s.d.'s in cell parameters are only used when they are defined by crystal symmetry. An approximate (isotropic) treatment of cell e.s.d.'s is used for estimating e.s.d.'s involving l.s. planes.
Refinement. Refinement of *F*^2^ against ALL reflections. The weighted *R*-factor *wR* and goodness of fit *S* are based on *F*^2^, conventional *R*-factors *R* are based on *F*, with *F* set to zero for negative *F*^2^. The threshold expression of *F*^2^ > σ(*F*^2^) is used only for calculating *R*-factors(gt) *etc*. and is not relevant to the choice of reflections for refinement. *R*-factors based on *F*^2^ are statistically about twice as large as those based on *F*, and *R*- factors based on ALL data will be even larger.

### Fractional atomic coordinates and isotropic or equivalent isotropic displacement parameters (Å^2^)

**Table:**

	*x*	*y*	*z*	*U*_iso_*/*U*_eq_	
C1	0.13185 (12)	0.5408 (3)	0.40308 (16)	0.0477 (6)	
H1	0.1436	0.4625	0.4326	0.057*	
C2	0.15571 (15)	0.6556 (3)	0.4506 (2)	0.0609 (8)	
H2	0.1832	0.6534	0.5105	0.073*	
C3	0.13835 (14)	0.7702 (3)	0.40838 (19)	0.0644 (8)	
H3	0.1536	0.8474	0.4394	0.077*	
C4	0.09731 (13)	0.7733 (3)	0.31771 (18)	0.0491 (7)	
C5	0.07771 (16)	0.8903 (3)	0.2685 (2)	0.0652 (9)	
H5	0.0920	0.9697	0.2971	0.078*	
C6	0.03932 (15)	0.8881 (3)	0.1826 (2)	0.0604 (8)	
H6	0.0278	0.9657	0.1524	0.072*	
C7	0.01567 (12)	0.7686 (2)	0.13628 (17)	0.0448 (6)	
C8	−0.02500 (12)	0.7611 (3)	0.04561 (18)	0.0517 (7)	
H8	−0.0379	0.8367	0.0131	0.062*	
C9	−0.04538 (13)	0.6440 (3)	0.00554 (18)	0.0510 (7)	
H9	−0.0719	0.6383	−0.0542	0.061*	
C10	−0.02569 (11)	0.5324 (3)	0.05582 (16)	0.0438 (6)	
H10	−0.0399	0.4524	0.0281	0.053*	
C11	0.03313 (11)	0.6513 (2)	0.18132 (16)	0.0352 (5)	
C12	0.07583 (11)	0.6531 (2)	0.27488 (16)	0.0362 (5)	
C13	0.16216 (11)	0.2707 (2)	0.25844 (15)	0.0393 (6)	
C14	0.22134 (11)	0.2278 (2)	0.27919 (16)	0.0407 (6)	
C15	0.26018 (13)	0.1335 (3)	0.34283 (19)	0.0518 (7)	
C16	0.31708 (14)	0.1016 (3)	0.3645 (2)	0.0701 (9)	
H16	0.3431	0.0400	0.4071	0.084*	
C17	0.33538 (17)	0.1594 (4)	0.3243 (3)	0.0817 (12)	
H17	0.3737	0.1362	0.3395	0.098*	
C18	0.29839 (16)	0.2517 (4)	0.2617 (3)	0.0750 (10)	
H18	0.3114	0.2908	0.2346	0.090*	
C19	0.24122 (14)	0.2856 (3)	0.2392 (2)	0.0558 (7)	
H19	0.2159	0.3480	0.1969	0.067*	
C20	−0.05515 (11)	0.2173 (2)	0.10588 (15)	0.0382 (5)	
C21	−0.11314 (11)	0.1442 (2)	0.03654 (15)	0.0361 (5)	
C22	−0.11635 (12)	0.0730 (2)	−0.02440 (15)	0.0432 (6)	
C23	−0.17163 (14)	0.0033 (3)	−0.08804 (16)	0.0565 (8)	
H23	−0.1743	−0.0454	−0.1285	0.068*	
C24	−0.22170 (13)	0.0073 (3)	−0.09029 (18)	0.0607 (8)	
H24	−0.2581	−0.0396	−0.1324	0.073*	
C25	−0.21937 (13)	0.0784 (3)	−0.03221 (18)	0.0576 (8)	
H25	−0.2541	0.0811	−0.0353	0.069*	
C26	−0.16526 (12)	0.1463 (2)	0.03120 (17)	0.0451 (6)	
H26	−0.1636	0.1944	0.0711	0.054*	
Cd1	0.057575 (8)	0.350425 (15)	0.231588 (11)	0.03562 (7)	
N1	0.09300 (9)	0.53845 (19)	0.31732 (12)	0.0372 (4)	
N2	0.01241 (8)	0.53497 (18)	0.14148 (12)	0.0361 (4)	
O1	0.13009 (8)	0.36071 (17)	0.20625 (12)	0.0517 (5)	
O2	0.14511 (8)	0.21631 (18)	0.29634 (11)	0.0497 (5)	
O3	0.24496 (10)	0.0733 (2)	0.38541 (14)	0.0733 (6)	
H3A	0.2083	0.0907	0.3625	0.110*	
O4	−0.00846 (8)	0.21515 (17)	0.10927 (12)	0.0527 (5)	
O5	0.05244 (8)	0.28218 (18)	0.34009 (12)	0.0516 (5)	
O6	−0.06878 (9)	0.06944 (18)	−0.02577 (12)	0.0582 (5)	
H6A	−0.0397	0.1157	0.0128	0.087*	

### Atomic displacement parameters (Å^2^)

**Table:**

	*U*^11^	*U*^22^	*U*^33^	*U*^12^	*U*^13^	*U*^23^
C1	0.0451 (14)	0.0544 (16)	0.0402 (15)	0.0009 (12)	0.0264 (12)	0.0023 (12)
C2	0.0598 (19)	0.074 (2)	0.0388 (15)	−0.0100 (15)	0.0276 (14)	−0.0083 (14)
C3	0.075 (2)	0.0561 (19)	0.0585 (19)	−0.0184 (16)	0.0419 (17)	−0.0227 (15)
C4	0.0547 (16)	0.0434 (15)	0.0540 (17)	−0.0046 (12)	0.0375 (14)	−0.0069 (12)
C5	0.083 (2)	0.0347 (15)	0.079 (2)	−0.0069 (15)	0.0531 (19)	−0.0092 (15)
C6	0.081 (2)	0.0320 (14)	0.074 (2)	0.0066 (14)	0.0534 (19)	0.0109 (14)
C7	0.0488 (14)	0.0373 (14)	0.0556 (16)	0.0067 (11)	0.0373 (13)	0.0082 (12)
C8	0.0521 (16)	0.0519 (17)	0.0555 (17)	0.0147 (13)	0.0370 (14)	0.0210 (14)
C9	0.0466 (15)	0.0615 (19)	0.0398 (15)	0.0083 (13)	0.0260 (13)	0.0099 (13)
C10	0.0407 (13)	0.0445 (14)	0.0398 (14)	0.0001 (11)	0.0235 (12)	0.0003 (11)
C11	0.0358 (12)	0.0347 (13)	0.0438 (14)	0.0014 (9)	0.0298 (12)	0.0025 (10)
C12	0.0361 (12)	0.0362 (13)	0.0426 (14)	−0.0019 (10)	0.0284 (11)	−0.0020 (10)
C13	0.0359 (13)	0.0407 (14)	0.0367 (13)	−0.0027 (10)	0.0217 (11)	−0.0052 (11)
C14	0.0380 (13)	0.0415 (14)	0.0453 (14)	−0.0039 (10)	0.0284 (12)	−0.0091 (11)
C15	0.0413 (15)	0.0504 (17)	0.0551 (17)	0.0027 (12)	0.0277 (14)	−0.0097 (13)
C16	0.0430 (16)	0.072 (2)	0.079 (2)	0.0128 (15)	0.0329 (17)	−0.0067 (18)
C17	0.052 (2)	0.089 (3)	0.110 (3)	−0.0032 (18)	0.055 (2)	−0.035 (2)
C18	0.080 (2)	0.082 (2)	0.106 (3)	−0.026 (2)	0.079 (2)	−0.032 (2)
C19	0.0632 (18)	0.0562 (18)	0.0655 (19)	−0.0087 (14)	0.0498 (16)	−0.0096 (14)
C20	0.0415 (13)	0.0274 (12)	0.0391 (13)	−0.0002 (10)	0.0233 (11)	0.0027 (10)
C21	0.0396 (13)	0.0301 (12)	0.0340 (12)	−0.0005 (9)	0.0219 (11)	0.0025 (9)
C22	0.0543 (15)	0.0336 (14)	0.0392 (14)	0.0038 (11)	0.0295 (12)	0.0044 (11)
C23	0.0702 (19)	0.0434 (16)	0.0355 (15)	−0.0025 (13)	0.0256 (14)	−0.0060 (12)
C24	0.0474 (17)	0.0550 (18)	0.0412 (16)	−0.0091 (13)	0.0121 (13)	−0.0018 (13)
C25	0.0417 (15)	0.0582 (18)	0.0542 (18)	−0.0056 (13)	0.0231 (14)	0.0044 (14)
C26	0.0446 (14)	0.0438 (15)	0.0421 (14)	0.0004 (11)	0.0261 (12)	0.0022 (11)
Cd1	0.03456 (11)	0.03147 (11)	0.03994 (12)	0.00079 (7)	0.02394 (9)	0.00196 (7)
N1	0.0367 (10)	0.0370 (11)	0.0375 (11)	0.0005 (8)	0.0240 (9)	0.0023 (9)
N2	0.0336 (10)	0.0365 (11)	0.0373 (11)	0.0004 (8)	0.0228 (9)	0.0009 (8)
O1	0.0432 (10)	0.0568 (12)	0.0545 (11)	0.0086 (8)	0.0316 (9)	0.0137 (9)
O2	0.0457 (10)	0.0594 (12)	0.0537 (11)	0.0101 (9)	0.0367 (9)	0.0135 (9)
O3	0.0634 (13)	0.0808 (16)	0.0761 (15)	0.0276 (11)	0.0457 (12)	0.0327 (13)
O4	0.0434 (10)	0.0479 (11)	0.0676 (13)	−0.0064 (8)	0.0367 (10)	−0.0098 (9)
O5	0.0585 (11)	0.0506 (11)	0.0498 (11)	0.0123 (9)	0.0373 (10)	0.0157 (9)
O6	0.0736 (13)	0.0545 (12)	0.0648 (13)	−0.0001 (10)	0.0532 (11)	−0.0074 (10)

### Geometric parameters (Å, °)

**Table:**

C1—N1	1.331 (3)	C16—H16	0.9300
C1—C2	1.394 (4)	C17—C18	1.373 (5)
C1—H1	0.9300	C17—H17	0.9300
C2—C3	1.353 (4)	C18—C19	1.388 (4)
C2—H2	0.9300	C18—H18	0.9300
C3—C4	1.407 (4)	C19—H19	0.9300
C3—H3	0.9300	C20—O5^i^	1.252 (3)
C4—C12	1.406 (3)	C20—O4	1.260 (3)
C4—C5	1.432 (4)	C20—C21	1.484 (3)
C5—C6	1.335 (5)	C21—C26	1.388 (4)
C5—H5	0.9300	C21—C22	1.399 (3)
C6—C7	1.426 (4)	C22—O6	1.348 (3)
C6—H6	0.9300	C22—C23	1.404 (4)
C7—C11	1.401 (3)	C23—C24	1.370 (4)
C7—C8	1.410 (4)	C23—H23	0.9300
C8—C9	1.358 (4)	C24—C25	1.362 (4)
C8—H8	0.9300	C24—H24	0.9300
C9—C10	1.395 (3)	C25—C26	1.378 (4)
C9—H9	0.9300	C25—H25	0.9300
C10—N2	1.331 (3)	C26—H26	0.9300
C10—H10	0.9300	Cd1—O2	2.3271 (16)
C11—N2	1.349 (3)	Cd1—N1	2.355 (2)
C11—C12	1.451 (4)	Cd1—N2	2.3606 (19)
C12—N1	1.355 (3)	Cd1—O4	2.3630 (18)
C13—O1	1.247 (3)	Cd1—O1	2.3993 (19)
C13—O2	1.275 (3)	Cd1—O5	2.4214 (18)
C13—C14	1.480 (3)	Cd1—O5^i^	2.4911 (18)
C14—C19	1.388 (4)	O3—H3A	0.8199
C14—C15	1.405 (4)	O5—C20^i^	1.252 (3)
C15—O3	1.345 (4)	O5—Cd1^i^	2.4911 (18)
C15—C16	1.382 (4)	O6—H6A	0.8200
C16—C17	1.357 (6)		
			
N1—C1—C2	122.8 (3)	O5^i^—C20—O4	119.8 (2)
N1—C1—H1	118.6	O5^i^—C20—C21	121.0 (2)
C2—C1—H1	118.6	O4—C20—C21	119.2 (2)
C3—C2—C1	119.1 (3)	C26—C21—C22	119.0 (2)
C3—C2—H2	120.4	C26—C21—C20	120.1 (2)
C1—C2—H2	120.4	C22—C21—C20	120.9 (2)
C2—C3—C4	120.4 (3)	O6—C22—C21	122.9 (2)
C2—C3—H3	119.8	O6—C22—C23	118.0 (2)
C4—C3—H3	119.8	C21—C22—C23	119.1 (3)
C12—C4—C3	116.8 (3)	C24—C23—C22	119.8 (3)
C12—C4—C5	119.5 (3)	C24—C23—H23	120.1
C3—C4—C5	123.7 (3)	C22—C23—H23	120.1
C6—C5—C4	121.4 (3)	C25—C24—C23	121.4 (3)
C6—C5—H5	119.3	C25—C24—H24	119.3
C4—C5—H5	119.3	C23—C24—H24	119.3
C5—C6—C7	121.0 (3)	C24—C25—C26	119.6 (3)
C5—C6—H6	119.5	C24—C25—H25	120.2
C7—C6—H6	119.5	C26—C25—H25	120.2
C11—C7—C8	117.0 (2)	C25—C26—C21	121.1 (3)
C11—C7—C6	119.8 (3)	C25—C26—H26	119.5
C8—C7—C6	123.2 (2)	C21—C26—H26	119.5
C9—C8—C7	120.2 (2)	O2—Cd1—N1	106.89 (7)
C9—C8—H8	119.9	O2—Cd1—N2	138.63 (6)
C7—C8—H8	119.9	N1—Cd1—N2	70.68 (7)
C8—C9—C10	118.7 (3)	O2—Cd1—O4	93.20 (6)
C8—C9—H9	120.7	N1—Cd1—O4	158.83 (6)
C10—C9—H9	120.7	N2—Cd1—O4	89.92 (6)
N2—C10—C9	123.1 (2)	O2—Cd1—O1	55.09 (6)
N2—C10—H10	118.4	N1—Cd1—O1	96.97 (7)
C9—C10—H10	118.4	N2—Cd1—O1	83.78 (6)
N2—C11—C7	122.8 (2)	O4—Cd1—O1	88.99 (7)
N2—C11—C12	117.8 (2)	O2—Cd1—O5	88.55 (6)
C7—C11—C12	119.4 (2)	N1—Cd1—O5	78.67 (7)
N1—C12—C4	122.7 (2)	N2—Cd1—O5	128.98 (6)
N1—C12—C11	118.5 (2)	O4—Cd1—O5	109.11 (7)
C4—C12—C11	118.8 (2)	O1—Cd1—O5	140.82 (6)
O1—C13—O2	120.2 (2)	O2—Cd1—O5^i^	126.96 (6)
O1—C13—C14	120.7 (2)	N1—Cd1—O5^i^	115.33 (6)
O2—C13—C14	119.0 (2)	N2—Cd1—O5^i^	86.73 (6)
C19—C14—C15	118.7 (3)	O4—Cd1—O5^i^	53.13 (6)
C19—C14—C13	120.3 (2)	O1—Cd1—O5^i^	140.94 (6)
C15—C14—C13	120.9 (2)	O5—Cd1—O5^i^	70.66 (7)
O3—C15—C16	117.8 (3)	C1—N1—C12	118.2 (2)
O3—C15—C14	122.7 (2)	C1—N1—Cd1	125.56 (17)
C16—C15—C14	119.5 (3)	C12—N1—Cd1	116.05 (15)
C17—C16—C15	120.7 (3)	C10—N2—C11	118.2 (2)
C17—C16—H16	119.6	C10—N2—Cd1	124.98 (16)
C15—C16—H16	119.6	C11—N2—Cd1	116.38 (15)
C16—C17—C18	121.2 (3)	C13—O1—Cd1	91.02 (15)
C16—C17—H17	119.4	C13—O2—Cd1	93.65 (15)
C18—C17—H17	119.4	C15—O3—H3A	109.4
C17—C18—C19	119.0 (3)	C20—O4—Cd1	96.43 (15)
C17—C18—H18	120.5	C20^i^—O5—Cd1	163.72 (17)
C19—C18—H18	120.5	C20^i^—O5—Cd1^i^	90.61 (15)
C14—C19—C18	120.9 (3)	Cd1—O5—Cd1^i^	99.62 (6)
C14—C19—H19	119.5	C22—O6—H6A	109.5
C18—C19—H19	119.5		
			
N1—C1—C2—C3	0.8 (5)	O1—Cd1—N1—C1	−99.78 (19)
C1—C2—C3—C4	−0.7 (5)	O5—Cd1—N1—C1	40.75 (19)
C2—C3—C4—C12	0.3 (4)	O5^i^—Cd1—N1—C1	102.81 (19)
C2—C3—C4—C5	−178.8 (3)	O2—Cd1—N1—C12	130.28 (16)
C12—C4—C5—C6	0.1 (5)	N2—Cd1—N1—C12	−6.16 (15)
C3—C4—C5—C6	179.1 (3)	O4—Cd1—N1—C12	−30.7 (3)
C4—C5—C6—C7	0.8 (5)	O1—Cd1—N1—C12	74.72 (16)
C5—C6—C7—C11	−0.4 (4)	O5—Cd1—N1—C12	−144.75 (17)
C5—C6—C7—C8	179.9 (3)	O5^i^—Cd1—N1—C12	−82.69 (17)
C11—C7—C8—C9	−0.2 (4)	C9—C10—N2—C11	−0.4 (4)
C6—C7—C8—C9	179.4 (3)	C9—C10—N2—Cd1	−172.1 (2)
C7—C8—C9—C10	0.5 (4)	C7—C11—N2—C10	0.7 (3)
C8—C9—C10—N2	−0.2 (4)	C12—C11—N2—C10	−178.6 (2)
C8—C7—C11—N2	−0.4 (4)	C7—C11—N2—Cd1	173.18 (18)
C6—C7—C11—N2	179.9 (2)	C12—C11—N2—Cd1	−6.2 (3)
C8—C7—C11—C12	178.9 (2)	O2—Cd1—N2—C10	84.4 (2)
C6—C7—C11—C12	−0.7 (4)	N1—Cd1—N2—C10	178.3 (2)
C3—C4—C12—N1	0.1 (4)	O4—Cd1—N2—C10	−10.32 (19)
C5—C4—C12—N1	179.3 (2)	O1—Cd1—N2—C10	78.67 (19)
C3—C4—C12—C11	179.7 (2)	O5—Cd1—N2—C10	−125.16 (18)
C5—C4—C12—C11	−1.2 (4)	O5^i^—Cd1—N2—C10	−63.38 (18)
N2—C11—C12—N1	0.4 (3)	O2—Cd1—N2—C11	−87.44 (18)
C7—C11—C12—N1	−178.9 (2)	N1—Cd1—N2—C11	6.44 (15)
N2—C11—C12—C4	−179.1 (2)	O4—Cd1—N2—C11	177.81 (16)
C7—C11—C12—C4	1.5 (4)	O1—Cd1—N2—C11	−93.21 (16)
O1—C13—C14—C19	−1.4 (4)	O5—Cd1—N2—C11	62.97 (18)
O2—C13—C14—C19	−179.7 (2)	O5^i^—Cd1—N2—C11	124.74 (16)
O1—C13—C14—C15	175.0 (2)	O2—C13—O1—Cd1	1.5 (2)
O2—C13—C14—C15	−3.3 (3)	C14—C13—O1—Cd1	−176.72 (19)
C19—C14—C15—O3	179.4 (3)	O2—Cd1—O1—C13	−0.88 (14)
C13—C14—C15—O3	2.9 (4)	N1—Cd1—O1—C13	104.88 (15)
C19—C14—C15—C16	0.6 (4)	N2—Cd1—O1—C13	174.48 (15)
C13—C14—C15—C16	−175.9 (2)	O4—Cd1—O1—C13	−95.49 (15)
O3—C15—C16—C17	−179.6 (3)	O5—Cd1—O1—C13	24.28 (19)
C14—C15—C16—C17	−0.8 (5)	O5^i^—Cd1—O1—C13	−108.56 (16)
C15—C16—C17—C18	0.5 (5)	O1—C13—O2—Cd1	−1.6 (2)
C16—C17—C18—C19	−0.1 (5)	C14—C13—O2—Cd1	176.70 (18)
C15—C14—C19—C18	−0.2 (4)	N1—Cd1—O2—C13	−85.86 (15)
C13—C14—C19—C18	176.3 (2)	N2—Cd1—O2—C13	−6.14 (19)
C17—C18—C19—C14	0.0 (5)	O4—Cd1—O2—C13	87.38 (15)
O5^i^—C20—C21—C26	−0.5 (3)	O1—Cd1—O2—C13	0.86 (13)
O4—C20—C21—C26	−179.4 (2)	O5—Cd1—O2—C13	−163.56 (15)
O5^i^—C20—C21—C22	179.2 (2)	O5^i^—Cd1—O2—C13	132.15 (14)
O4—C20—C21—C22	0.3 (3)	O5^i^—C20—O4—Cd1	0.7 (2)
C26—C21—C22—O6	177.6 (2)	C21—C20—O4—Cd1	179.66 (17)
C20—C21—C22—O6	−2.1 (3)	O2—Cd1—O4—C20	134.91 (14)
C26—C21—C22—C23	−1.4 (3)	N1—Cd1—O4—C20	−63.3 (2)
C20—C21—C22—C23	178.9 (2)	N2—Cd1—O4—C20	−86.37 (15)
O6—C22—C23—C24	−178.3 (2)	O1—Cd1—O4—C20	−170.14 (15)
C21—C22—C23—C24	0.8 (4)	O5—Cd1—O4—C20	45.33 (15)
C22—C23—C24—C25	0.4 (4)	O5^i^—Cd1—O4—C20	−0.40 (13)
C23—C24—C25—C26	−1.1 (4)	O2—Cd1—O5—C20^i^	−34.5 (6)
C24—C25—C26—C21	0.4 (4)	N1—Cd1—O5—C20^i^	−142.0 (6)
C22—C21—C26—C25	0.8 (4)	N2—Cd1—O5—C20^i^	164.6 (6)
C20—C21—C26—C25	−179.5 (2)	O4—Cd1—O5—C20^i^	58.4 (6)
C2—C1—N1—C12	−0.4 (4)	O1—Cd1—O5—C20^i^	−54.9 (6)
C2—C1—N1—Cd1	174.0 (2)	O5^i^—Cd1—O5—C20^i^	95.8 (6)
C4—C12—N1—C1	−0.1 (4)	O2—Cd1—O5—Cd1^i^	−162.70 (7)
C11—C12—N1—C1	−179.6 (2)	N1—Cd1—O5—Cd1^i^	89.75 (7)
C4—C12—N1—Cd1	−174.99 (19)	N2—Cd1—O5—Cd1^i^	36.35 (10)
C11—C12—N1—Cd1	5.5 (3)	O4—Cd1—O5—Cd1^i^	−69.82 (8)
O2—Cd1—N1—C1	−44.2 (2)	O1—Cd1—O5—Cd1^i^	176.89 (7)
N2—Cd1—N1—C1	179.3 (2)	O5^i^—Cd1—O5—Cd1^i^	−32.44 (8)
O4—Cd1—N1—C1	154.77 (19)		

Symmetry codes: (i) −*x*, *y*, −*z*+1/2.

### Hydrogen-bond geometry (Å, °)

**Table:**

*D*—H···*A*	*D*—H	H···*A*	*D*···*A*	*D*—H···*A*
O6—H6A···O4	0.82	1.86	2.579 (3)	146
O3—H3A···O2	0.82	1.87	2.576 (2)	143
